# Crystal structure of penta­potassium di­hydrogen nona­vanadato(V)platinate(IV) nona­hydrate

**DOI:** 10.1107/S2056989015009135

**Published:** 2015-05-20

**Authors:** Hea-Chung Joo, Uk Lee

**Affiliations:** a Department of Chemistry, Pukyong National University, 599-1 Daeyeon 3-dong, Nam-gu, Busan 608-737, Republic of Korea

**Keywords:** crystal structure, nona­vanadoplatinate(IV) polyanion, heteropolyanions, hydrogen bonding

## Abstract

The title compound, K_5_[H_2_PtV_9_O_28_]·9H_2_O, containing the nona­vanado­platinate(IV) polyanion, was obtained by hydro­thermal reaction at pH = 4.2. The polyanion has approximate *mm*2 (*C*_2*v*_) symmetry. The two platinum-bound μ_2_-O atoms are protonated in the polyanion. The heteropolyanions form inversion-generated dimers, {[H_2_PtV_9_O_28_]_2_}^10−^, held together by μ_2_-O–H⋯μ_2_-O and μ_2_-O–H⋯μ_3_-O hydrogen bonds. All K^+^ cations are located on general positions of the space group *P*-1.

## Abstract 2

The polyanion in K_5_[H_2_PtV_9_O_28_]·9H_2_O has approximate *mm*2 (*C*_2*v*_) symmetry. The two platinum-bound μ_2_-O atoms are protonated in the polyanion. The heteropolyanions form inversion-generated dimers, {[H_2_PtV_9_O_28_]_2_}^10−^, held together by μ_2_-O—H⋯μ_2_-O and μ_2_-O—H⋯μ_3_-O hydrogen bonds.

## Structural commentary   

Two heteropolyanions that belong to the deca­vanadate structure system (Lee, 2006 ▸) have recently been reported: the tellurium derivative [H_*n*_TeV_9_O_28_]^(5−*n*)^ (*n* = 1 and 2), described by Konaka *et al.* (2011 ▸), and the platinum heteropoly­oxido­vanadate, [H_2_PtV_9_O_28_]^5−^, reported by our group in the form of its sodium salt, Na_5_[H_2_PtV_9_O_28_]·21H_2_O (Lee *et al.*, 2008 ▸) and a guanidinium salt, (CH_6_N_3_)_5_[H_2_PtV_9_O_28_] (Joo *et al.*, 2011 ▸). The Te heteroatom of the [H_*n*_TeV_9_O_28_]^(5−*n*)^ polyanion was located on two sites (corresponding to the Pt1 and V4 sites in the title compound) by disorder. However, the Pt atom does not show any disorder in three [H_2_PtV_9_O_28_]^5−^ polyanions. We herein report the structure of the title compound because it could contribute to our knowledge of the structural character­istics of the [H_2_PtV_9_O_28_]^5−^ polyanion.

**Figure 1:**
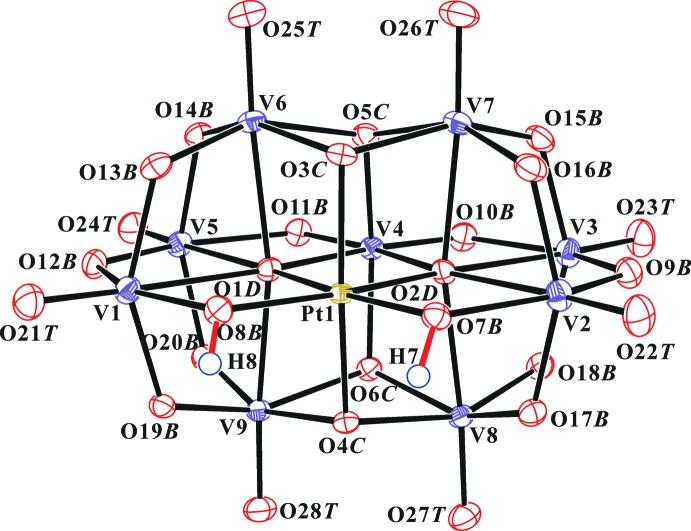
The mol­ecular structure of the heteropolyanion in the title compound showing the atom-numbering scheme. Displacement ellipsoids are drawn at the 50% probability level. H atoms are presented as small spheres of arbitrary radius.

**Figure 2:**
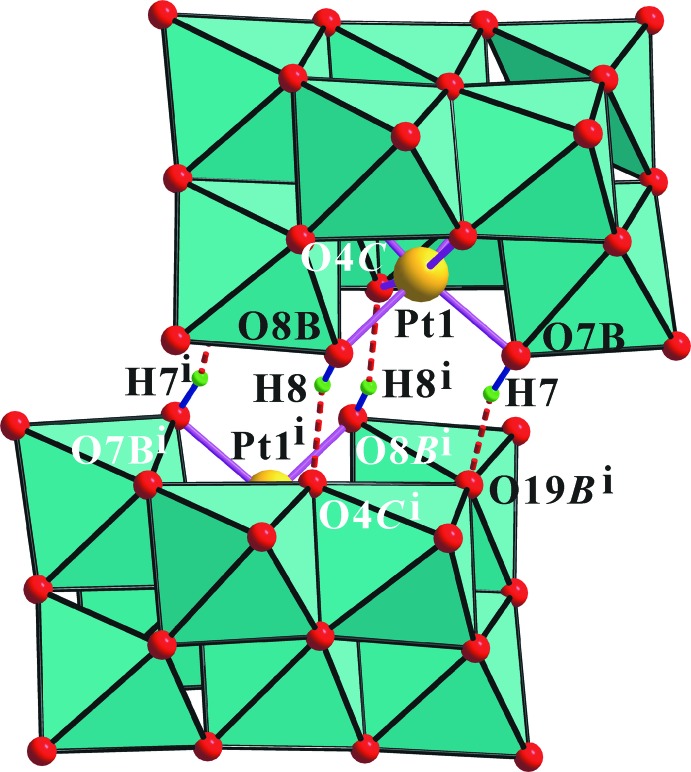
Polyhedral view of the inter-anion hydrogen bonds (dotted lines) in the crystal structure of the title compound. [Symmetry code: (i) −*x*, −*y* + 1, −*z*.]

Fig. 1 ▸ shows the structure of the heteropolyanion in the title compound. The O atoms of the clusters were designated as O*T* (terminal, V=O), O*B* (bridging, μ_2_-O), O*C* (μ_3_-O), and O*D* (μ_4_-O). All atoms in the polyanions are located in general positions. The protonated O*B* atoms in the polyanions were identified by the locations in the difference Fourier maps of the H atoms bound to atoms O7*B* and O8*B* and local structural features, as seen previously in sodium and guanidinium salts, respectively. The geometry of the anion agrees well with that in sodium and guanidinium salts. The nine [VO_6_] octa­hedra in the polyanion are distorted [range of V—O distances = 1.596 (3)–2.403 (3) Å], while the [PtO_6_] octa­hedron is relatively regular [Pt—O = 1.985 (3)–2.036 (3) Å]. The two platinum bound μ_2_-O atoms are protonated in the polyanion. These protons are particularly important in the solid state as they lead to the formation of a dimeric assembly, {[H_2_PtV_9_O_28_]_2_}^10−^, through each of the two μ_2_-O7*B*–H7⋯μ_2_-O19*B* and μ_2_-O8*B*–H8⋯μ_3_-O4*C* inter­anion hydrogen bonds (Fig. 2 ▸ and Table 1 ▸).

The K^+^ ions are variously coordinated by O atoms as [K1(O*B*)(O*T*)_2_(O*W*)_5_]^+^ in the range 2.725 (5)–3.351 (6) Å, [K2(O*B*)_2_(O*T*)_3_(O*W*)_3_]^+^ in the range 2.722 (4)–3.156 (5) Å, [K3(O*B*)(O*T*)_4_(O*W*)_4_]^+^ in the range 2.844 (4)–3.151 (3) Å, [K4(O*B*)(O*T*)_2_(O*W*)_4_]^+^ in the range 2.733 (5)–3.284 (7) Å, and [K5(O*B*)_2_(O*T*)_2_(O*W*)_3_]^+^ in the range 2.734 (6)–2.996 (4) Å. The bond-valence sums (BVS; Brown & Altermatt, 1985 ▸; Brese & O’Keeffe, 1991 ▸) for the K1, K2, K3, K4, and K5 cations are 0.99, 1.12,1.04, 0.81, and 1.10 v.u, respectively (total v.u. = 5.06).

The polyanion dimers are three-dimensionally linked *via* K⋯O*T* and K⋯O*B* inter­actions. All water mol­ecules form hydrogen bonds with polyanions except for the O9*W* water mol­ecule (Table 1 ▸).

## Synthesis and crystallization   

Single crystals of the title compound were obtained in the same way as the sodium salt reported by Lee *et al.* (2008 ▸) using K_2_Pt(OH)_6_ and KVO_3_.

## Refinement   

Crystal data, data collection and structure refinement details are summarized in Table 2 ▸. Atoms H7 and H8, bound to μ_2_-O7*B* and μ_2_-O8*B*, respectively, of the polyanion were found in a difference Fourier map and were freely refined. The H atoms of the O6*W* mol­ecule were positioned geometrically and refined using a riding model (*SHELXL2014* command HFIX 23), with O—H = 0.97 Å and *U*_iso_(H) = 1.5 *U*_eq_(O). All other water H atoms were refined with distance restraints of O—H = 0.85 (3) Å and H*A*⋯H*B* = 1.35 (3) Å using DFIX, and were included in the refinement with *U*_iso_(H) = 1.5*U*_eq_(O). The unusually short μ_2_-O17*B*⋯terminal-O21*T* ^i^ distance of 2.949 (5) Å (symmetry code as in Fig. 2 ▸.) is caused by the neighboring hydrogen bonds between the polyanions of the dimer as shown in Fig. 2 ▸. The highest peak in the difference map is 0.95 Å from K4 and the largest hole is 0.92 Å from Pt1.

## Supplementary Material

Crystal structure: contains datablock(s) I. DOI: 10.1107/S2056989015009135/vn2093sup1.cif

Structure factors: contains datablock(s) I. DOI: 10.1107/S2056989015009135/vn2093Isup2.hkl

CCDC reference: 1400654

Additional supporting information:  crystallographic information; 3D view; checkCIF report

## Figures and Tables

**Table 1:** Hydrogen-bond geometry (, )

*D*H*A*	*D*H	H*A*	*D* *A*	*D*H*A*
O7*B*H7O19*B* ^i^	0.90(8)	1.86(8)	2.738(4)	164(8)
O8*B*H8O4*C* ^i^	0.77(6)	1.90(6)	2.645(5)	161(6)
O1*W*H1*B*O9*B*	0.83(3)	2.50(6)	3.127(6)	133(7)
O1*W*H1*A*O8*W*	0.84(3)	2.10(3)	2.930(9)	170(8)
O2*W*H2*A*O15*B*	0.87(3)	1.88(3)	2.728(6)	166(8)
O2*W*H2*B*O11*B* ^ii^	0.86(3)	2.23(5)	2.975(6)	145(8)
O3*W*H3*A*O3*C* ^iii^	0.84(3)	1.83(3)	2.673(5)	177(10)
O3*W*H3*B*O7*W*	0.83(3)	2.09(5)	2.875(7)	158(8)
O4*W*H4*A*O13*B* ^i^	0.83(3)	1.88(3)	2.680(5)	162(7)
O4*W*H4*B*O14*B* ^iv^	0.83(3)	2.10(4)	2.850(5)	151(7)
O5*W*H5*A*O2*W* ^v^	0.87(3)	1.84(3)	2.710(8)	175(8)
O5*W*H5*B*O24*T* ^vi^	0.84(3)	2.26(5)	2.972(6)	142(7)
O6*W*H6*A*O5*C* ^iv^	0.97	1.81	2.755(5)	163
O6*W*H6*B*O10*B* ^v^	0.97	2.04	2.755(5)	129
O7*W*H7*A*O7*B* ^iii^	0.85(3)	2.07(4)	2.891(5)	163(8)
O7*W*H7*B*O6*C* ^v^	0.84(3)	2.27(6)	2.885(5)	130(6)
O8*W*H8*A*O5*W* ^v^	0.86(3)	1.98(5)	2.795(7)	159(10)
O8*W*H8*B*O19*B* ^vii^	0.86(3)	2.22(4)	3.031(6)	156(8)

Symmetry codes: (i) 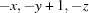; (ii) 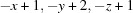; (iii) 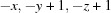; (iv) 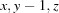; (v) 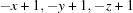; (vi) 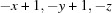; (vii) 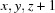.

**Table 2:** Experimental details

Crystal data	
Chemical formula	K_5_[H_2_PtV_9_O_28_]9H_2_O
*M*_r_	1461.21
Crystal system, space group	Triclinic, *P* 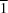
Temperature (K)	298
*a*, *b*, *c* ()	10.1663(7), 12.8350(7), 13.615(2)
, , ()	103.734(5), 106.193(6), 92.480(4)
*V* (^3^)	1645.8(3)
*Z*	2
Radiation type	Mo *K*
(mm^1^)	7.42
Crystal size (mm)	0.21 0.19 0.17

Data collection	
Diffractometer	Stoe Stadi4
Absorption correction	Empirical (using intensity measurements) (*X-SHAPE*; Stoe Cie,1996 ▸)
*T*_min_, *T*_max_	0.301, 0.378
No. of measured, independent and observed [*I* > 2(*I*)] reflections	6797, 6797, 6242
(sin /)_max_ (^1^)	0.628

Refinement	
*R*[*F* ^2^ > 2(*F* ^2^)], *wR*(*F* ^2^), *S*	0.028, 0.066, 1.10
No. of reflections	6797
No. of parameters	526
No. of restraints	25
H-atom treatment	H atoms treated by a mixture of independent and constrained refinement
_max_, _min_ (e ^3^)	1.30, 1.46

Computer programs: *STADI4* and *X-RED* (Stoe Cie, 1996 ▸), *SHELXS2014* (Sheldrick, 2008 ▸), *SHELXL2014* (Sheldrick, 2015 ▸), *ORTEP-3 for Windows* (Farrugia, 2012 ▸) and *DIAMOND* (Brandenburg, 1998 ▸).

## supplementary crystallographic information

### Crystal data

**Table:**

K_5_[H_2_PtV_9_O_28_]·9H_2_O	*Z* = 2
*M**_r_* = 1461.21	*F*(000) = 1392
Triclinic, *P*1	*D*_x_ = 2.949 Mg m^−^^3^
*a* = 10.1663 (7) Å	Mo *K*α radiation, λ = 0.71069 Å
*b* = 12.8350 (7) Å	Cell parameters from 30 reflections
*c* = 13.615 (2) Å	θ = 9.6–10.5°
α = 103.734 (5)°	µ = 7.42 mm^−^^1^
β = 106.193 (6)°	*T* = 298 K
γ = 92.480 (4)°	Block, dark brown
*V* = 1645.8 (3) Å^3^	0.21 × 0.19 × 0.17 mm

### Data collection

**Table:**

Stoe Stadi4 diffractometer	*R*_int_ = 0.0000
Radiation source: fine-focus sealed tube	θ_max_ = 26.5°, θ_min_ = 1.6°
ω/2–θ scans	*h* = −12→12
Absorption correction: empirical (using intensity measurements) (*X-SHAPE*; Stoe & Cie,1996)	*k* = −16→15
*T*_min_ = 0.301, *T*_max_ = 0.378	*l* = 0→17
6797 measured reflections	3 standard reflections every 60 min
6797 independent reflections	intensity decay: 2.5%
6242 reflections with *I* > 2σ(*I*)	

### Refinement

**Table:**

Refinement on *F*^2^	Secondary atom site location: difference Fourier map
Least-squares matrix: full	Hydrogen site location: difference Fourier map
*R*[*F*^2^ > 2σ(*F*^2^)] = 0.028	H atoms treated by a mixture of independent and constrained refinement
*wR*(*F*^2^) = 0.066	*w* = 1/[σ^2^(*F*_o_^2^) + (0.0277*P*)^2^ + 4.5476*P*] where *P* = (*F*_o_^2^ + 2*F*_c_^2^)/3
*S* = 1.10	(Δ/σ)_max_ < 0.001
6797 reflections	Δρ_max_ = 1.30 e Å^−^^3^
526 parameters	Δρ_min_ = −1.46 e Å^−^^3^
25 restraints	Extinction correction: *SHELXL2014* (Sheldrick, 2015), Fc^*^=kFc[1+0.001xFc^2^λ^3^/sin(2θ)]^-1/4^
Primary atom site location: structure-invariant direct methods	Extinction coefficient: 0.00505 (13)

### Special details

**Table:**

Geometry. All esds (except the esd in the dihedral angle between two l.s. planes) are estimated using the full covariance matrix. The cell esds are taken into account individually in the estimation of esds in distances, angles and torsion angles; correlations between esds in cell parameters are only used when they are defined by crystal symmetry. An approximate (isotropic) treatment of cell esds is used for estimating esds involving l.s. planes.

### Fractional atomic coordinates and isotropic or equivalent isotropic displacement parameters (Å^2^)

**Table:**

	*x*	*y*	*z*	*U*_iso_*/*U*_eq_	
Pt1	0.07476 (2)	0.66204 (2)	0.15945 (2)	0.01102 (6)	
V1	0.08091 (8)	0.74803 (6)	−0.03864 (6)	0.01675 (16)	
V2	0.05610 (8)	0.57644 (6)	0.35482 (6)	0.01798 (16)	
V3	0.36393 (8)	0.67118 (6)	0.46862 (6)	0.01860 (17)	
V4	0.38427 (7)	0.76066 (6)	0.27828 (6)	0.01375 (15)	
V5	0.38558 (8)	0.84633 (6)	0.08300 (6)	0.01680 (16)	
V6	0.15181 (8)	0.90837 (6)	0.19135 (6)	0.01625 (16)	
V7	0.14522 (8)	0.82358 (6)	0.38572 (6)	0.01838 (17)	
V8	0.28571 (8)	0.50931 (6)	0.24210 (6)	0.01521 (16)	
V9	0.30124 (8)	0.59746 (6)	0.04752 (6)	0.01441 (15)	
K1	0.19325 (16)	0.94505 (11)	0.81002 (11)	0.0433 (3)	
K2	0.32640 (13)	0.27185 (9)	0.99666 (11)	0.0329 (3)	
K3	0.29033 (13)	0.19821 (10)	0.25987 (10)	0.0353 (3)	
K4	0.1688 (2)	0.15936 (14)	0.5300 (2)	0.0819 (7)	
K5	0.33393 (14)	0.48579 (12)	0.61615 (13)	0.0439 (3)	
O1D	0.2234 (3)	0.7454 (2)	0.1318 (2)	0.0148 (6)	
O2D	0.2133 (3)	0.6723 (2)	0.2981 (2)	0.0148 (6)	
O3C	0.0321 (3)	0.8071 (2)	0.2320 (2)	0.0156 (6)	
O4C	0.1590 (3)	0.5298 (2)	0.1039 (2)	0.0137 (6)	
O5C	0.2793 (3)	0.8770 (2)	0.3209 (2)	0.0165 (6)	
O6C	0.3986 (3)	0.6174 (2)	0.2014 (2)	0.0147 (6)	
O7B	−0.0627 (3)	0.5793 (3)	0.2053 (3)	0.0159 (6)	
H7	−0.086 (8)	0.519 (6)	0.152 (6)	0.07 (2)*	
O8B	−0.0508 (3)	0.6659 (3)	0.0160 (3)	0.0168 (7)	
H8	−0.067 (6)	0.609 (5)	−0.023 (5)	0.021 (15)*	
O9B	0.2105 (3)	0.5994 (3)	0.4730 (3)	0.0202 (7)	
O10B	0.4757 (3)	0.7538 (3)	0.3997 (3)	0.0207 (7)	
O11B	0.4856 (3)	0.8317 (3)	0.2321 (3)	0.0179 (7)	
O12B	0.2409 (3)	0.8257 (3)	−0.0358 (3)	0.0197 (7)	
O13B	0.0439 (3)	0.8680 (2)	0.0548 (3)	0.0182 (7)	
O14B	0.3033 (3)	0.9526 (2)	0.1585 (3)	0.0174 (6)	
O15B	0.2871 (3)	0.8035 (3)	0.4919 (3)	0.0215 (7)	
O16B	0.0221 (3)	0.7196 (3)	0.3937 (3)	0.0204 (7)	
O17B	0.1402 (3)	0.4691 (2)	0.2815 (3)	0.0176 (6)	
O18B	0.4015 (3)	0.5523 (3)	0.3774 (3)	0.0184 (7)	
O19B	0.1590 (3)	0.6178 (2)	−0.0635 (2)	0.0158 (6)	
O20B	0.4224 (3)	0.7028 (3)	0.0424 (3)	0.0179 (7)	
O21T	−0.0224 (4)	0.7505 (3)	−0.1503 (3)	0.0270 (8)	
O22T	−0.0570 (4)	0.5099 (3)	0.3842 (3)	0.0257 (8)	
O23T	0.4743 (4)	0.6778 (3)	0.5812 (3)	0.0287 (8)	
O24T	0.5067 (3)	0.9142 (3)	0.0618 (3)	0.0238 (7)	
O25T	0.0976 (4)	1.0199 (3)	0.2403 (3)	0.0245 (7)	
O26T	0.0934 (4)	0.9340 (3)	0.4384 (3)	0.0301 (8)	
O27T	0.3379 (4)	0.3970 (3)	0.1941 (3)	0.0237 (7)	
O28T	0.3493 (4)	0.4863 (3)	−0.0055 (3)	0.0234 (7)	
O1W	0.1060 (6)	0.7552 (4)	0.6420 (4)	0.0514 (12)	
H1A	0.154 (8)	0.721 (6)	0.681 (5)	0.077*	
H1B	0.133 (8)	0.750 (7)	0.589 (4)	0.077*	
O2W	0.3763 (6)	0.9426 (4)	0.6891 (4)	0.0618 (15)	
H2A	0.347 (9)	0.908 (6)	0.623 (3)	0.093*	
H2B	0.428 (8)	0.997 (5)	0.688 (6)	0.093*	
O3W	0.2228 (5)	0.1440 (4)	0.7752 (6)	0.0660 (17)	
H3A	0.143 (5)	0.161 (7)	0.775 (8)	0.099*	
H3B	0.268 (7)	0.197 (5)	0.770 (8)	0.099*	
O4W	0.2138 (4)	0.0846 (3)	0.0168 (4)	0.0356 (9)	
H4A	0.132 (4)	0.097 (5)	0.007 (6)	0.053*	
H4B	0.224 (6)	0.029 (4)	0.038 (6)	0.053*	
O5W	0.5107 (5)	0.1657 (4)	0.1656 (4)	0.0495 (12)	
H5A	0.550 (8)	0.130 (5)	0.210 (4)	0.074*	
H5B	0.481 (8)	0.120 (5)	0.106 (3)	0.074*	
O6W	0.3583 (5)	0.0913 (4)	0.4279 (4)	0.0552 (13)	
H6A	0.3463	0.0134	0.4006	0.083*	
H6B	0.4520	0.1148	0.4737	0.083*	
O7W	0.3045 (5)	0.3465 (4)	0.7411 (4)	0.0471 (12)	
H7A	0.242 (5)	0.367 (6)	0.769 (6)	0.071*	
H7B	0.373 (5)	0.394 (5)	0.774 (6)	0.071*	
O8W	0.3022 (6)	0.6482 (5)	0.7759 (4)	0.0634 (15)	
H8A	0.375 (6)	0.694 (6)	0.797 (6)	0.095*	
H8B	0.287 (9)	0.645 (7)	0.834 (4)	0.095*	
O9W	0.1770 (7)	0.3233 (4)	0.4164 (5)	0.0629 (15)	
H9A	0.265 (3)	0.341 (8)	0.444 (7)	0.094*	
H9B	0.159 (8)	0.357 (7)	0.368 (5)	0.094*	

### Atomic displacement parameters (Å^2^)

**Table:**

	*U*^11^	*U*^22^	*U*^33^	*U*^12^	*U*^13^	*U*^23^
Pt1	0.00977 (9)	0.00941 (9)	0.01224 (9)	−0.00006 (5)	0.00238 (6)	0.00097 (6)
V1	0.0165 (4)	0.0157 (4)	0.0161 (4)	0.0005 (3)	0.0017 (3)	0.0046 (3)
V2	0.0159 (4)	0.0202 (4)	0.0177 (4)	−0.0023 (3)	0.0051 (3)	0.0053 (3)
V3	0.0183 (4)	0.0199 (4)	0.0148 (4)	−0.0021 (3)	0.0010 (3)	0.0047 (3)
V4	0.0120 (3)	0.0126 (3)	0.0144 (4)	−0.0016 (3)	0.0020 (3)	0.0019 (3)
V5	0.0162 (4)	0.0151 (4)	0.0194 (4)	−0.0013 (3)	0.0058 (3)	0.0050 (3)
V6	0.0182 (4)	0.0106 (3)	0.0187 (4)	0.0017 (3)	0.0048 (3)	0.0022 (3)
V7	0.0231 (4)	0.0151 (4)	0.0160 (4)	0.0024 (3)	0.0076 (3)	0.0000 (3)
V8	0.0168 (4)	0.0121 (3)	0.0162 (4)	0.0025 (3)	0.0041 (3)	0.0036 (3)
V9	0.0158 (4)	0.0120 (3)	0.0157 (4)	0.0015 (3)	0.0060 (3)	0.0026 (3)
K1	0.0556 (9)	0.0407 (7)	0.0386 (7)	0.0112 (6)	0.0207 (7)	0.0109 (6)
K2	0.0380 (7)	0.0222 (6)	0.0465 (7)	0.0055 (5)	0.0257 (6)	0.0078 (5)
K3	0.0327 (6)	0.0355 (7)	0.0352 (7)	−0.0054 (5)	0.0075 (5)	0.0090 (5)
K4	0.1103 (16)	0.0426 (9)	0.1207 (18)	0.0171 (10)	0.0879 (15)	0.0091 (10)
K5	0.0328 (7)	0.0496 (8)	0.0667 (10)	0.0148 (6)	0.0246 (7)	0.0346 (7)
O1D	0.0146 (15)	0.0120 (14)	0.0172 (16)	−0.0012 (12)	0.0047 (12)	0.0033 (12)
O2D	0.0153 (15)	0.0137 (15)	0.0126 (15)	−0.0033 (12)	0.0026 (12)	0.0010 (12)
O3C	0.0157 (15)	0.0128 (15)	0.0167 (16)	0.0019 (12)	0.0059 (12)	−0.0007 (12)
O4C	0.0136 (14)	0.0106 (14)	0.0153 (15)	0.0004 (11)	0.0036 (12)	0.0017 (12)
O5C	0.0169 (15)	0.0136 (15)	0.0161 (15)	0.0002 (12)	0.0028 (12)	0.0012 (12)
O6C	0.0128 (14)	0.0135 (15)	0.0153 (15)	−0.0002 (12)	0.0012 (12)	0.0030 (12)
O7B	0.0159 (15)	0.0150 (16)	0.0159 (16)	0.0004 (12)	0.0051 (13)	0.0022 (13)
O8B	0.0147 (15)	0.0162 (17)	0.0156 (16)	−0.0006 (13)	−0.0005 (13)	0.0031 (14)
O9B	0.0221 (17)	0.0206 (17)	0.0169 (16)	−0.0022 (13)	0.0055 (13)	0.0040 (13)
O10B	0.0194 (16)	0.0191 (16)	0.0205 (17)	−0.0027 (13)	0.0026 (13)	0.0044 (13)
O11B	0.0142 (15)	0.0180 (16)	0.0204 (17)	−0.0025 (12)	0.0038 (13)	0.0049 (13)
O12B	0.0243 (17)	0.0174 (16)	0.0179 (16)	−0.0010 (13)	0.0055 (14)	0.0069 (13)
O13B	0.0162 (15)	0.0129 (15)	0.0236 (17)	0.0007 (12)	0.0031 (13)	0.0050 (13)
O14B	0.0187 (16)	0.0124 (15)	0.0210 (17)	−0.0007 (12)	0.0061 (13)	0.0041 (13)
O15B	0.0273 (18)	0.0197 (17)	0.0141 (16)	−0.0007 (14)	0.0062 (14)	−0.0017 (13)
O16B	0.0226 (17)	0.0192 (16)	0.0204 (17)	0.0025 (13)	0.0107 (14)	0.0018 (13)
O17B	0.0190 (16)	0.0142 (15)	0.0185 (16)	−0.0015 (12)	0.0049 (13)	0.0039 (13)
O18B	0.0169 (15)	0.0173 (16)	0.0185 (16)	0.0016 (12)	0.0009 (13)	0.0052 (13)
O19B	0.0167 (15)	0.0144 (15)	0.0138 (15)	0.0029 (12)	0.0022 (12)	0.0014 (12)
O20B	0.0180 (16)	0.0178 (16)	0.0200 (17)	0.0031 (13)	0.0085 (13)	0.0054 (13)
O21T	0.0298 (19)	0.0255 (18)	0.0220 (18)	0.0016 (15)	0.0007 (15)	0.0075 (15)
O22T	0.0233 (18)	0.0306 (19)	0.0266 (19)	−0.0029 (15)	0.0109 (15)	0.0106 (15)
O23T	0.0287 (19)	0.033 (2)	0.0186 (18)	−0.0072 (16)	−0.0024 (15)	0.0079 (15)
O24T	0.0224 (17)	0.0224 (17)	0.0281 (19)	−0.0033 (14)	0.0091 (15)	0.0086 (15)
O25T	0.0245 (18)	0.0164 (16)	0.032 (2)	0.0048 (14)	0.0081 (15)	0.0041 (14)
O26T	0.039 (2)	0.0213 (18)	0.029 (2)	0.0061 (16)	0.0154 (17)	−0.0020 (15)
O27T	0.0264 (18)	0.0180 (17)	0.0283 (19)	0.0074 (14)	0.0085 (15)	0.0077 (14)
O28T	0.0278 (18)	0.0164 (16)	0.0277 (19)	0.0037 (14)	0.0130 (15)	0.0034 (14)
O1W	0.066 (3)	0.050 (3)	0.041 (3)	0.008 (2)	0.025 (3)	0.007 (2)
O2W	0.080 (4)	0.056 (3)	0.035 (3)	−0.024 (3)	0.017 (3)	−0.010 (2)
O3W	0.028 (2)	0.058 (3)	0.126 (5)	0.013 (2)	0.028 (3)	0.044 (4)
O4W	0.028 (2)	0.035 (2)	0.054 (3)	0.0107 (17)	0.0133 (19)	0.027 (2)
O5W	0.052 (3)	0.041 (3)	0.049 (3)	−0.008 (2)	0.013 (2)	0.004 (2)
O6W	0.051 (3)	0.034 (2)	0.056 (3)	−0.011 (2)	0.003 (2)	−0.014 (2)
O7W	0.030 (2)	0.043 (3)	0.078 (4)	0.0107 (19)	0.023 (2)	0.025 (2)
O8W	0.080 (4)	0.066 (4)	0.042 (3)	−0.016 (3)	0.028 (3)	0.002 (3)
O9W	0.095 (4)	0.051 (3)	0.056 (3)	0.025 (3)	0.029 (3)	0.028 (3)

### Geometric parameters (Å, º)

**Table:**

Pt1—V6	3.1213 (8)	V8—O27T	1.612 (3)
Pt1—V8	3.1262 (8)	V8—O17B	1.803 (3)
Pt1—V9	3.1359 (8)	V8—O18B	1.826 (3)
Pt1—V7	3.1480 (9)	V8—O6C	2.033 (3)
Pt1—V4	3.1566 (8)	V8—O4C	2.045 (3)
Pt1—V2	3.1574 (9)	V8—O2D	2.278 (3)
V1—V5	3.1204 (11)	V9—O28T	1.601 (3)
V1—V6	3.1801 (11)	V9—O20B	1.816 (3)
V1—V9	3.1802 (11)	V9—O19B	1.856 (3)
V2—V3	3.1217 (11)	V9—O6C	2.001 (3)
V2—V7	3.1566 (11)	V9—O4C	2.064 (3)
V2—V8	3.1767 (11)	V9—O1D	2.270 (3)
V2—V4	4.4982 (11)	K1—O3W^i^	2.725 (5)
V3—V4	3.1255 (11)	K1—O2W	2.804 (6)
V3—V8	3.1476 (11)	K1—O1W	2.827 (5)
V3—V7	3.1731 (11)	K1—O12B^ii^	2.830 (3)
V4—V5	3.1120 (11)	K1—O4W^iii^	2.903 (5)
V4—V9	3.1960 (11)	K1—O25T^iv^	2.924 (4)
V4—V7	3.2078 (11)	K1—O24T^v^	3.267 (4)
V4—V8	3.2162 (10)	K1—O5W^vi^	3.351 (6)
V5—V9	3.1578 (10)	K2—O4W^ii^	2.722 (4)
V5—V6	3.1654 (11)	K2—O27T^ii^	2.753 (4)
V6—V7	3.1068 (12)	K2—O28T^ii^	2.760 (3)
V8—V9	3.1566 (11)	K2—O20B^vi^	2.772 (3)
Pt1—O1D	1.985 (3)	K2—O8B^vii^	2.917 (3)
Pt1—O2D	1.986 (3)	K2—O3W	2.936 (7)
Pt1—O4C	2.015 (3)	K2—O24T^vi^	3.069 (4)
Pt1—O3C	2.017 (3)	K2—O5W^ii^	3.156 (5)
Pt1—O8B	2.027 (3)	K3—O23T^vi^	2.844 (4)
Pt1—O7B	2.036 (3)	K3—O25T^viii^	2.860 (4)
V1—O21T	1.598 (3)	K3—O5W	2.876 (5)
V1—O12B	1.857 (3)	K3—O6W	2.878 (6)
V1—O13B	1.876 (3)	K3—O9W	2.890 (6)
V1—O19B	1.880 (3)	K3—O21T^ix^	2.904 (4)
V1—O8B	2.064 (3)	K3—O27T	2.960 (4)
V1—O1D	2.377 (3)	K3—O4W	3.139 (5)
V2—O22T	1.596 (3)	K3—O14B^viii^	3.151 (3)
V2—O9B	1.862 (3)	K4—O6W	2.733 (5)
V2—O16B	1.863 (3)	K4—O16B^vii^	2.806 (4)
V2—O17B	1.885 (3)	K4—O26T^viii^	2.841 (4)
V2—O7B	2.067 (3)	K4—O9W	2.904 (5)
V2—O2D	2.374 (3)	K4—O26T^vii^	3.055 (4)
V3—O23T	1.608 (3)	K4—O7W	3.187 (6)
V3—O9B	1.800 (3)	K4—O3W	3.284 (7)
V3—O18B	1.853 (3)	K5—O8W	2.734 (6)
V3—O15B	1.895 (3)	K5—O18B^vi^	2.736 (3)
V3—O10B	2.064 (3)	K5—O9B	2.764 (4)
V3—O2D	2.403 (3)	K5—O7W	2.803 (5)
V4—O10B	1.681 (3)	K5—O22T^vii^	2.817 (4)
V4—O11B	1.685 (3)	K5—O9W	2.982 (7)
V4—O6C	1.921 (3)	K5—O23T	2.996 (4)
V4—O5C	1.943 (3)	O7B—H7	0.90 (8)
V4—O2D	2.148 (3)	O8B—H8	0.77 (6)
V4—O1D	2.158 (3)	O1W—H1A	0.84 (3)
V5—O24T	1.607 (3)	O1W—H1B	0.83 (3)
V5—O12B	1.813 (3)	O2W—H2A	0.87 (3)
V5—O20B	1.878 (3)	O2W—H2B	0.86 (3)
V5—O14B	1.879 (3)	O3W—H3A	0.84 (3)
V5—O11B	2.058 (3)	O3W—H3B	0.83 (3)
V5—O1D	2.381 (3)	O4W—H4A	0.83 (3)
V6—O25T	1.617 (3)	O4W—H4B	0.83 (3)
V6—O13B	1.812 (3)	O5W—H5A	0.87 (3)
V6—O14B	1.824 (3)	O5W—H5B	0.84 (3)
V6—O3C	2.014 (3)	O6W—H6A	0.9700
V6—O5C	2.015 (3)	O6W—H6B	0.9700
V6—O1D	2.284 (3)	O7W—H7A	0.85 (3)
V7—O26T	1.610 (3)	O7W—H7B	0.84 (3)
V7—O15B	1.812 (3)	O8W—H8A	0.86 (3)
V7—O16B	1.832 (3)	O8W—H8B	0.86 (3)
V7—O5C	1.997 (3)	O9W—H9A	0.87 (3)
V7—O3C	2.043 (3)	O9W—H9B	0.85 (3)
V7—O2D	2.269 (3)		
			
O1D—Pt1—O2D	84.61 (12)	Pt1—V4—V8	58.745 (19)
O1D—Pt1—O4C	85.94 (12)	V9—V4—V8	58.98 (2)
O2D—Pt1—O4C	85.94 (12)	V7—V4—V8	89.47 (3)
O1D—Pt1—O3C	85.44 (12)	V4—V5—V1	92.76 (3)
O2D—Pt1—O3C	84.79 (12)	V4—V5—V9	61.29 (2)
O4C—Pt1—O3C	167.89 (12)	V1—V5—V9	60.86 (2)
O1D—Pt1—O8B	88.49 (13)	V4—V5—V6	62.06 (2)
O2D—Pt1—O8B	172.89 (13)	V1—V5—V6	60.78 (3)
O4C—Pt1—O8B	95.27 (13)	V9—V5—V6	91.58 (3)
O3C—Pt1—O8B	92.99 (13)	V4—V5—Pt1	46.338 (17)
O1D—Pt1—O7B	173.32 (13)	V1—V5—Pt1	46.428 (18)
O2D—Pt1—O7B	88.75 (12)	V9—V5—Pt1	45.928 (17)
O4C—Pt1—O7B	94.36 (12)	V6—V5—Pt1	45.659 (17)
O3C—Pt1—O7B	93.22 (13)	V7—V6—Pt1	60.72 (2)
O8B—Pt1—O7B	98.12 (13)	V7—V6—V5	118.88 (3)
O1D—Pt1—V6	46.91 (9)	Pt1—V6—V5	87.85 (2)
O2D—Pt1—V6	88.79 (9)	V7—V6—V1	120.95 (3)
O4C—Pt1—V6	132.85 (9)	Pt1—V6—V1	60.23 (2)
O3C—Pt1—V6	39.21 (9)	V5—V6—V1	58.91 (2)
O8B—Pt1—V6	85.25 (10)	V6—V7—V3	120.25 (3)
O7B—Pt1—V6	132.36 (9)	Pt1—V7—V3	87.79 (2)
O1D—Pt1—V8	89.65 (9)	V2—V7—V3	59.10 (2)
O2D—Pt1—V8	46.63 (9)	V6—V7—V4	61.64 (2)
O4C—Pt1—V8	40.00 (9)	Pt1—V7—V4	59.55 (2)
O3C—Pt1—V8	131.42 (9)	V2—V7—V4	89.94 (3)
O8B—Pt1—V8	135.22 (10)	V3—V7—V4	58.66 (2)
O7B—Pt1—V8	86.34 (9)	Pt1—V8—V3	88.63 (2)
V6—Pt1—V8	123.63 (2)	Pt1—V8—V9	59.88 (2)
O1D—Pt1—V9	46.16 (9)	V3—V8—V9	119.00 (3)
O2D—Pt1—V9	89.07 (9)	V3—V8—V2	59.15 (2)
O4C—Pt1—V9	40.35 (9)	V9—V8—V2	120.00 (3)
O3C—Pt1—V9	131.59 (9)	V3—V8—V4	58.82 (2)
O8B—Pt1—V9	87.36 (10)	V9—V8—V4	60.19 (2)
O7B—Pt1—V9	134.69 (9)	V2—V8—V4	89.43 (3)
V6—Pt1—V9	92.83 (2)	Pt1—V9—V8	59.58 (2)
V8—Pt1—V9	60.54 (2)	Pt1—V9—V5	87.73 (2)
O1D—Pt1—V7	88.61 (9)	V8—V9—V5	119.42 (3)
O2D—Pt1—V7	45.87 (9)	Pt1—V9—V1	60.07 (2)
O4C—Pt1—V7	131.81 (9)	V8—V9—V1	119.64 (3)
O3C—Pt1—V7	39.46 (9)	V5—V9—V1	58.99 (2)
O8B—Pt1—V7	132.43 (10)	Pt1—V9—V4	59.80 (2)
O7B—Pt1—V7	86.22 (9)	V8—V9—V4	60.83 (2)
V6—Pt1—V7	59.41 (2)	V5—V9—V4	58.65 (2)
V8—Pt1—V7	92.22 (2)	V1—V9—V4	90.08 (3)
V9—Pt1—V7	122.21 (2)	O3W^i^—K1—O2W	71.45 (18)
O1D—Pt1—V4	42.45 (9)	O3W^i^—K1—O1W	122.0 (2)
O2D—Pt1—V4	42.16 (9)	O2W—K1—O1W	73.89 (15)
O4C—Pt1—V4	84.02 (8)	O3W^i^—K1—O12B^ii^	145.45 (18)
O3C—Pt1—V4	83.88 (9)	O2W—K1—O12B^ii^	118.85 (16)
O8B—Pt1—V4	130.94 (9)	O1W—K1—O12B^ii^	92.12 (13)
O7B—Pt1—V4	130.91 (9)	O3W^i^—K1—O4W^iii^	78.75 (18)
V6—Pt1—V4	62.06 (2)	O2W—K1—O4W^iii^	128.72 (14)
V8—Pt1—V4	61.58 (2)	O1W—K1—O4W^iii^	155.55 (14)
V9—Pt1—V4	61.05 (2)	O12B^ii^—K1—O4W^iii^	69.55 (10)
V7—Pt1—V4	61.17 (2)	O3W^i^—K1—O25T^iv^	82.50 (12)
O1D—Pt1—V2	133.30 (9)	O2W—K1—O25T^iv^	129.92 (16)
O2D—Pt1—V2	48.70 (9)	O1W—K1—O25T^iv^	85.91 (13)
O4C—Pt1—V2	90.75 (9)	O12B^ii^—K1—O25T^iv^	106.98 (11)
O3C—Pt1—V2	88.98 (9)	O4W^iii^—K1—O25T^iv^	84.34 (11)
O8B—Pt1—V2	138.14 (9)	O3W^i^—K1—O24T^v^	65.13 (14)
O7B—Pt1—V2	40.05 (9)	O2W—K1—O24T^v^	65.37 (12)
V6—Pt1—V2	119.45 (2)	O1W—K1—O24T^v^	133.36 (13)
V8—Pt1—V2	60.73 (2)	O12B^ii^—K1—O24T^v^	88.44 (10)
V9—Pt1—V2	121.27 (2)	O4W^iii^—K1—O24T^v^	64.45 (10)
V7—Pt1—V2	60.08 (2)	O25T^iv^—K1—O24T^v^	138.04 (10)
V4—Pt1—V2	90.86 (2)	O3W^i^—K1—O5W^vi^	110.45 (14)
O21T—V1—O12B	102.27 (17)	O2W—K1—O5W^vi^	51.31 (16)
O21T—V1—O13B	102.73 (17)	O1W—K1—O5W^vi^	79.54 (14)
O12B—V1—O13B	90.13 (14)	O12B^ii^—K1—O5W^vi^	67.77 (12)
O21T—V1—O19B	105.72 (16)	O4W^iii^—K1—O5W^vi^	106.58 (12)
O12B—V1—O19B	91.05 (14)	O25T^iv^—K1—O5W^vi^	164.20 (12)
O13B—V1—O19B	150.57 (14)	O24T^v^—K1—O5W^vi^	57.73 (10)
O21T—V1—O8B	99.44 (16)	O4W^ii^—K2—O27T^ii^	96.79 (13)
O12B—V1—O8B	158.14 (14)	O4W^ii^—K2—O28T^ii^	157.70 (12)
O13B—V1—O8B	82.64 (14)	O27T^ii^—K2—O28T^ii^	71.43 (10)
O19B—V1—O8B	85.45 (14)	O4W^ii^—K2—O20B^vi^	124.36 (11)
O21T—V1—O1D	176.60 (16)	O27T^ii^—K2—O20B^vi^	110.65 (11)
O12B—V1—O1D	80.42 (12)	O28T^ii^—K2—O20B^vi^	77.91 (10)
O13B—V1—O1D	75.06 (12)	O4W^ii^—K2—O8B^vii^	82.79 (11)
O19B—V1—O1D	76.16 (12)	O27T^ii^—K2—O8B^vii^	73.02 (10)
O8B—V1—O1D	77.79 (12)	O28T^ii^—K2—O8B^vii^	75.73 (10)
O22T—V2—O9B	103.62 (17)	O20B^vi^—K2—O8B^vii^	150.46 (10)
O22T—V2—O16B	104.45 (17)	O4W^ii^—K2—O3W	78.19 (15)
O9B—V2—O16B	91.43 (15)	O27T^ii^—K2—O3W	162.23 (13)
O22T—V2—O17B	104.04 (17)	O28T^ii^—K2—O3W	107.12 (13)
O9B—V2—O17B	88.97 (14)	O20B^vi^—K2—O3W	85.81 (12)
O16B—V2—O17B	150.56 (14)	O8B^vii^—K2—O3W	89.37 (12)
O22T—V2—O7B	97.56 (16)	O4W^ii^—K2—O24T^vi^	69.29 (11)
O9B—V2—O7B	158.78 (14)	O27T^ii^—K2—O24T^vi^	128.86 (11)
O16B—V2—O7B	84.50 (14)	O28T^ii^—K2—O24T^vi^	132.88 (10)
O17B—V2—O7B	84.59 (13)	O20B^vi^—K2—O24T^vi^	55.63 (9)
O22T—V2—O2D	175.77 (15)	O8B^vii^—K2—O24T^vi^	145.32 (10)
O9B—V2—O2D	80.53 (13)	O3W—K2—O24T^vi^	65.65 (11)
O16B—V2—O2D	76.07 (13)	O4W^ii^—K2—O5W^ii^	63.16 (12)
O17B—V2—O2D	74.97 (12)	O27T^ii^—K2—O5W^ii^	72.57 (12)
O7B—V2—O2D	78.27 (11)	O28T^ii^—K2—O5W^ii^	126.96 (12)
O23T—V3—O9B	104.10 (17)	O20B^vi^—K2—O5W^ii^	79.90 (12)
O23T—V3—O18B	104.12 (17)	O8B^vii^—K2—O5W^ii^	127.23 (12)
O9B—V3—O18B	92.55 (15)	O3W—K2—O5W^ii^	118.55 (13)
O23T—V3—O15B	103.56 (18)	O24T^vi^—K2—O5W^ii^	57.03 (11)
O9B—V3—O15B	90.80 (15)	O23T^vi^—K3—O25T^viii^	136.47 (11)
O18B—V3—O15B	150.37 (14)	O23T^vi^—K3—O5W	75.77 (13)
O23T—V3—O10B	101.92 (16)	O25T^viii^—K3—O5W	120.42 (12)
O9B—V3—O10B	153.94 (14)	O23T^vi^—K3—O6W	72.84 (12)
O18B—V3—O10B	82.41 (14)	O25T^viii^—K3—O6W	64.17 (12)
O15B—V3—O10B	81.76 (14)	O5W—K3—O6W	104.30 (15)
O23T—V3—O2D	174.90 (16)	O23T^vi^—K3—O9W	76.16 (15)
O9B—V3—O2D	80.92 (13)	O25T^viii^—K3—O9W	87.40 (14)
O18B—V3—O2D	76.28 (12)	O5W—K3—O9W	150.12 (16)
O15B—V3—O2D	75.21 (12)	O6W—K3—O9W	76.75 (14)
O10B—V3—O2D	73.04 (12)	O23T^vi^—K3—O21T^ix^	133.79 (11)
O10B—V4—O11B	108.05 (16)	O25T^viii^—K3—O21T^ix^	74.44 (10)
O10B—V4—O6C	98.10 (15)	O5W—K3—O21T^ix^	123.14 (14)
O11B—V4—O6C	99.13 (15)	O6W—K3—O21T^ix^	128.44 (13)
O10B—V4—O5C	98.12 (15)	O9W—K3—O21T^ix^	71.85 (14)
O11B—V4—O5C	96.71 (15)	O23T^vi^—K3—O27T	74.05 (10)
O6C—V4—O5C	152.55 (13)	O25T^viii^—K3—O27T	146.54 (11)
O10B—V4—O2D	87.63 (14)	O5W—K3—O27T	73.96 (12)
O11B—V4—O2D	164.23 (14)	O6W—K3—O27T	146.13 (12)
O6C—V4—O2D	79.74 (12)	O9W—K3—O27T	88.67 (13)
O5C—V4—O2D	78.95 (12)	O21T^ix^—K3—O27T	72.77 (10)
O10B—V4—O1D	164.37 (14)	O23T^vi^—K3—O4W	136.36 (11)
O11B—V4—O1D	87.58 (14)	O25T^viii^—K3—O4W	78.53 (11)
O6C—V4—O1D	79.38 (12)	O5W—K3—O4W	61.86 (12)
O5C—V4—O1D	79.03 (12)	O6W—K3—O4W	125.62 (12)
O2D—V4—O1D	76.74 (12)	O9W—K3—O4W	141.67 (15)
O24T—V5—O12B	104.93 (17)	O21T^ix^—K3—O4W	70.07 (10)
O24T—V5—O20B	103.26 (16)	O27T—K3—O4W	84.26 (10)
O12B—V5—O20B	91.36 (15)	O23T^vi^—K3—O14B^viii^	119.30 (11)
O24T—V5—O14B	103.76 (16)	O25T^viii^—K3—O14B^viii^	53.38 (9)
O12B—V5—O14B	91.43 (15)	O5W—K3—O14B^viii^	67.26 (11)
O20B—V5—O14B	151.11 (14)	O6W—K3—O14B^viii^	72.00 (11)
O24T—V5—O11B	100.00 (16)	O9W—K3—O14B^viii^	137.58 (13)
O12B—V5—O11B	155.07 (14)	O21T^ix^—K3—O14B^viii^	106.80 (10)
O20B—V5—O11B	82.61 (14)	O27T—K3—O14B^viii^	132.52 (10)
O14B—V5—O11B	82.82 (14)	O4W—K3—O14B^viii^	53.88 (9)
O24T—V5—O1D	173.91 (15)	O6W—K4—O16B^vii^	162.73 (15)
O12B—V5—O1D	81.14 (13)	O6W—K4—O26T^viii^	73.88 (13)
O20B—V5—O1D	75.91 (12)	O16B^vii^—K4—O26T^viii^	118.24 (13)
O14B—V5—O1D	76.13 (12)	O6W—K4—O9W	78.81 (17)
O11B—V5—O1D	73.92 (11)	O16B^vii^—K4—O9W	83.95 (15)
O25T—V6—O13B	103.58 (16)	O26T^viii^—K4—O9W	126.27 (17)
O25T—V6—O14B	103.90 (16)	O6W—K4—O26T^vii^	138.56 (13)
O13B—V6—O14B	94.04 (15)	O16B^vii^—K4—O26T^vii^	54.50 (10)
O25T—V6—O3C	97.90 (16)	O26T^viii^—K4—O26T^vii^	64.75 (12)
O13B—V6—O3C	91.22 (14)	O9W—K4—O26T^vii^	124.14 (16)
O14B—V6—O3C	155.66 (14)	O6W—K4—O7W	111.55 (14)
O25T—V6—O5C	102.60 (16)	O16B^vii^—K4—O7W	65.92 (11)
O13B—V6—O5C	152.25 (14)	O26T^viii^—K4—O7W	146.61 (15)
O14B—V6—O5C	88.47 (14)	O9W—K4—O7W	86.49 (16)
O3C—V6—O5C	76.20 (13)	O26T^vii^—K4—O7W	104.46 (12)
O25T—V6—O1D	175.51 (15)	O6W—K4—O3W	118.81 (16)
O13B—V6—O1D	78.63 (13)	O16B^vii^—K4—O3W	74.21 (12)
O14B—V6—O1D	79.71 (12)	O26T^viii^—K4—O3W	95.01 (14)
O3C—V6—O1D	78.07 (12)	O9W—K4—O3W	138.71 (17)
O5C—V6—O1D	74.63 (12)	O26T^vii^—K4—O3W	68.97 (12)
O26T—V7—O15B	103.92 (18)	O7W—K4—O3W	52.73 (14)
O26T—V7—O16B	102.74 (18)	O8W—K5—O18B^vi^	116.68 (16)
O15B—V7—O16B	95.34 (16)	O8W—K5—O9B	88.96 (16)
O26T—V7—O5C	102.03 (17)	O18B^vi^—K5—O9B	115.20 (11)
O15B—V7—O5C	89.99 (14)	O8W—K5—O7W	86.37 (17)
O16B—V7—O5C	152.57 (14)	O18B^vi^—K5—O7W	94.58 (12)
O26T—V7—O3C	98.14 (17)	O9B—K5—O7W	148.49 (12)
O15B—V7—O3C	155.92 (14)	O8W—K5—O22T^vii^	67.49 (15)
O16B—V7—O3C	89.04 (14)	O18B^vi^—K5—O22T^vii^	170.27 (11)
O5C—V7—O3C	75.93 (13)	O9B—K5—O22T^vii^	72.81 (10)
O26T—V7—O2D	175.00 (16)	O7W—K5—O22T^vii^	76.63 (12)
O15B—V7—O2D	80.25 (13)	O8W—K5—O9W	142.73 (18)
O16B—V7—O2D	79.42 (13)	O18B^vi^—K5—O9W	100.56 (14)
O5C—V7—O2D	75.00 (12)	O9B—K5—O9W	73.02 (12)
O3C—V7—O2D	77.31 (12)	O7W—K5—O9W	92.42 (15)
O27T—V8—O17B	104.28 (16)	O22T^vii^—K5—O9W	75.98 (14)
O27T—V8—O18B	102.86 (16)	O8W—K5—O23T	78.72 (14)
O17B—V8—O18B	94.12 (15)	O18B^vi^—K5—O23T	71.86 (10)
O27T—V8—O6C	101.85 (16)	O9B—K5—O23T	55.51 (10)
O17B—V8—O6C	152.21 (14)	O7W—K5—O23T	152.01 (14)
O18B—V8—O6C	89.12 (14)	O22T^vii^—K5—O23T	117.87 (11)
O27T—V8—O4C	98.38 (15)	O9W—K5—O23T	113.75 (13)
O17B—V8—O4C	91.54 (13)	Pt1—O1D—V4	99.17 (13)
O18B—V8—O4C	155.90 (13)	Pt1—O1D—V9	94.73 (12)
O6C—V8—O4C	75.36 (12)	V4—O1D—V9	92.37 (12)
O27T—V8—O2D	175.32 (15)	Pt1—O1D—V6	93.70 (12)
O17B—V8—O2D	78.97 (13)	V4—O1D—V6	93.47 (12)
O18B—V8—O2D	80.09 (13)	V9—O1D—V6	168.86 (15)
O6C—V8—O2D	74.42 (12)	Pt1—O1D—V1	92.47 (12)
O4C—V8—O2D	78.04 (11)	V4—O1D—V1	168.36 (15)
O28T—V9—O20B	105.26 (16)	V9—O1D—V1	86.35 (11)
O28T—V9—O19B	102.34 (16)	V6—O1D—V1	86.01 (10)
O20B—V9—O19B	94.63 (14)	Pt1—O1D—V5	174.40 (16)
O28T—V9—O6C	102.11 (16)	V4—O1D—V5	86.40 (11)
O20B—V9—O6C	90.31 (14)	V9—O1D—V5	85.49 (10)
O19B—V9—O6C	152.78 (13)	V6—O1D—V5	85.42 (10)
O28T—V9—O4C	96.57 (15)	V1—O1D—V5	81.96 (10)
O20B—V9—O4C	156.16 (14)	Pt1—O2D—V4	99.47 (13)
O19B—V9—O4C	89.88 (13)	Pt1—O2D—V7	95.20 (12)
O6C—V9—O4C	75.62 (12)	V4—O2D—V7	93.10 (11)
O28T—V9—O1D	174.17 (15)	Pt1—O2D—V8	94.04 (12)
O20B—V9—O1D	80.02 (13)	V4—O2D—V8	93.16 (12)
O19B—V9—O1D	79.42 (12)	V7—O2D—V8	167.84 (15)
O6C—V9—O1D	75.11 (12)	Pt1—O2D—V2	92.35 (11)
O4C—V9—O1D	77.82 (11)	V4—O2D—V2	168.17 (15)
V5—V1—Pt1	87.93 (2)	V7—O2D—V2	85.63 (11)
V5—V1—V6	60.31 (2)	V8—O2D—V2	86.10 (10)
Pt1—V1—V6	58.97 (2)	Pt1—O2D—V3	173.86 (16)
V5—V1—V9	60.15 (2)	V4—O2D—V3	86.57 (10)
Pt1—V1—V9	59.27 (2)	V7—O2D—V3	85.52 (10)
V6—V1—V9	90.89 (3)	V8—O2D—V3	84.47 (10)
V5—V1—V4	43.54 (2)	V2—O2D—V3	81.61 (10)
Pt1—V1—V4	44.385 (15)	V6—O3C—Pt1	101.52 (13)
V6—V1—V4	45.82 (2)	V6—O3C—V7	99.96 (13)
V9—V1—V4	45.099 (19)	Pt1—O3C—V7	101.69 (14)
V3—V2—V7	60.71 (3)	Pt1—O4C—V8	100.70 (13)
V3—V2—Pt1	88.53 (3)	Pt1—O4C—V9	100.45 (13)
V7—V2—Pt1	59.81 (2)	V8—O4C—V9	100.37 (13)
V3—V2—V8	59.96 (3)	V4—O5C—V7	109.01 (15)
V7—V2—V8	91.11 (3)	V4—O5C—V6	109.70 (15)
Pt1—V2—V8	59.15 (2)	V7—O5C—V6	101.51 (14)
V3—V2—V4	43.98 (2)	V4—O6C—V9	109.15 (14)
V7—V2—V4	45.49 (2)	V4—O6C—V8	108.85 (15)
Pt1—V2—V4	44.562 (14)	V9—O6C—V8	103.00 (13)
V8—V2—V4	45.641 (19)	Pt1—O7B—V2	100.62 (13)
V3—V2—V1	89.54 (2)	Pt1—O8B—V1	101.22 (14)
V7—V2—V1	59.98 (2)	V4—O10B—V3	112.76 (17)
Pt1—V2—V1	1.113 (10)	V4—O11B—V5	112.09 (16)
V8—V2—V1	60.11 (2)	V5—O12B—V1	116.48 (17)
V4—V2—V1	45.564 (14)	V6—O13B—V1	119.14 (17)
V2—V3—V4	92.11 (3)	V6—O14B—V5	117.51 (16)
V2—V3—V8	60.89 (3)	V7—O15B—V3	117.70 (17)
V4—V3—V8	61.69 (3)	V7—O16B—V2	117.32 (17)
V2—V3—V7	60.19 (3)	V8—O17B—V2	118.96 (16)
V4—V3—V7	61.23 (2)	V8—O18B—V3	117.66 (17)
V8—V3—V7	91.34 (3)	V9—O19B—V1	116.70 (16)
V5—V4—Pt1	88.17 (2)	V9—O20B—V5	117.45 (17)
V3—V4—Pt1	88.48 (2)	H1A—O1W—H1B	109 (5)
V5—V4—V9	60.06 (2)	H2A—O2W—H2B	102 (4)
V3—V4—V9	118.47 (3)	H3A—O3W—H3B	107 (5)
Pt1—V4—V9	59.155 (19)	H4A—O4W—H4B	109 (4)
V5—V4—V7	117.42 (3)	H5A—O5W—H5B	106 (4)
V3—V4—V7	60.12 (3)	H6A—O6W—H6B	109.4
Pt1—V4—V7	59.28 (2)	H7A—O7W—H7B	105 (4)
V9—V4—V7	118.44 (3)	H8A—O8W—H8B	103 (4)
V5—V4—V8	118.99 (3)	H9A—O9W—H9B	101 (4)
V3—V4—V8	59.49 (2)		

Symmetry codes: (i) *x*, *y*+1, *z*; (ii) *x*, *y*, *z*+1; (iii) *x*, *y*+1, *z*+1; (iv) −*x*, −*y*+2, −*z*+1; (v) −*x*+1, −*y*+2, −*z*+1; (vi) −*x*+1, −*y*+1, −*z*+1; (vii) −*x*, −*y*+1, −*z*+1; (viii) *x*, *y*−1, *z*; (ix) −*x*, −*y*+1, −*z*.

### Hydrogen-bond geometry (Å, º)

**Table:**

*D*—H···*A*	*D*—H	H···*A*	*D*···*A*	*D*—H···*A*
O7*B*—H7···O19*B*^ix^	0.90 (8)	1.86 (8)	2.738 (4)	164 (8)
O8*B*—H8···O4*C*^ix^	0.77 (6)	1.90 (6)	2.645 (5)	161 (6)
O1*W*—H1*B*···O9*B*	0.83 (3)	2.50 (6)	3.127 (6)	133 (7)
O1*W*—H1*A*···O8*W*	0.84 (3)	2.10 (3)	2.930 (9)	170 (8)
O2*W*—H2*A*···O15*B*	0.87 (3)	1.88 (3)	2.728 (6)	166 (8)
O2*W*—H2*B*···O11*B*^v^	0.86 (3)	2.23 (5)	2.975 (6)	145 (8)
O3*W*—H3*A*···O3*C*^vii^	0.84 (3)	1.83 (3)	2.673 (5)	177 (10)
O3*W*—H3*B*···O7*W*	0.83 (3)	2.09 (5)	2.875 (7)	158 (8)
O4*W*—H4*A*···O13*B*^ix^	0.83 (3)	1.88 (3)	2.680 (5)	162 (7)
O4*W*—H4*B*···O14*B*^viii^	0.83 (3)	2.10 (4)	2.850 (5)	151 (7)
O5*W*—H5*A*···O2*W*^vi^	0.87 (3)	1.84 (3)	2.710 (8)	175 (8)
O5*W*—H5*B*···O24*T*^x^	0.84 (3)	2.26 (5)	2.972 (6)	142 (7)
O6*W*—H6*A*···O5*C*^viii^	0.97	1.81	2.755 (5)	163
O6*W*—H6*B*···O10*B*^vi^	0.97	2.04	2.755 (5)	129
O7*W*—H7*A*···O7*B*^vii^	0.85 (3)	2.07 (4)	2.891 (5)	163 (8)
O7*W*—H7*B*···O6*C*^vi^	0.84 (3)	2.27 (6)	2.885 (5)	130 (6)
O8*W*—H8*A*···O5*W*^vi^	0.86 (3)	1.98 (5)	2.795 (7)	159 (10)
O8*W*—H8*B*···O19*B*^ii^	0.86 (3)	2.22 (4)	3.031 (6)	156 (8)

Symmetry codes: (ii) *x*, *y*, *z*+1; (v) −*x*+1, −*y*+2, −*z*+1; (vi) −*x*+1, −*y*+1, −*z*+1; (vii) −*x*, −*y*+1, −*z*+1; (viii) *x*, *y*−1, *z*; (ix) −*x*, −*y*+1, −*z*; (x) −*x*+1, −*y*+1, −*z*.
